# Editorial: A Review of Micronutrients and the Immune System—Working in Harmony to Reduce the Risk of Infection

**DOI:** 10.3390/nu13114180

**Published:** 2021-11-22

**Authors:** Ascensión Marcos

**Affiliations:** Department of Metabolism and Nutrition, Institute of Food Science, Technology and Nutrition (ICTAN), Spanish National Research Council (CSIC), President of the International Society for Immunonutrition (ISIN), 28040 Madrid, Spain; amarcos@ictan.csic.es

The emergence of the SARS-CoV virus in December 2019 saw the beginning of an unprecedented pandemic that represents the most significant public health problem in recent memory. Initial public health strategies were mainly reactive, including social distancing and hygienic practices, therapeutic drugs to improve patient outcomes, and vaccine development and distribution, all aimed at preventing the spread of the highly-infective virus. However, there is a need for additional strategies to prevent severe COVID-19-related complications, and to ensure a proper immune response following vaccination [1,2]. One outcome of the pandemic has been the increasing public awareness of the role of the immune system as the first line of defense against external pathogens, as well as the importance of adequate nutrition in maintaining strong immune defenses [1,2,3,4]. This is why nutrition should be the most important prevention factor to count on. Indeed, a high-quality diet has recently been shown to reduce both the risk and severity of COVID-19, particularly in areas with higher socioeconomic deprivation [5]. This ensures that nutritional support is a safe and cost-effective strategy that could be implemented to promote optimal immune function [1,2].

As highlighted by Gombart et al. [6], nutrient intake is a critical factor that influences the strength of the immune response. This review summarized the key roles of multiple micronutrients in supporting immune defenses against viruses and bacteria, including vitamins A, D, C, E, B6, and B12, folate, zinc, iron, copper, and selenium. These micronutrients are fundamental for immune cell function, protection against oxidative stress (e.g., zinc, vitamin C), and a healthy inflammatory response, while some also have anti-viral efefcts (e.g., zinc, vitamin D, selenium). Gombart and coworkers discussed how a low micronutrient intake and status impair the immune response, thereby increasing susceptibility to infections and enabling them to become more severe. This situation may be prevented and even reversed by micronutrient repletion. A strength of the review is that it illustrates the fact that micronutrients do not work alone; instead, it provides a holistic view of their complementary roles in maintaining a healthy immune system by following the journey of a pathogen as it attempts to enter the human body. In this novel way, the various sequential components of the immune response against infections are discussed, along with the fundamental and synergistic roles that micronutrients play throughout the entire complex immune response. A summary of the key data on micronutrient status and the risk of infection, as well as on the effects of micronutrient supplementation on resistance to infection, is also provided and clearly suggests that multiple micronutrient supplementation may help to improve immune function and resistance to infection.

Published in January 2020, the article by Gombart et al. preceded the surge of interest triggered by the COVID-19 pandemic in the interactions between nutritional status and immune function. Since then, numerous scientific reviews describing the possible beneficial roles of micronutrients in the physiopathology of COVID-19 (including their anti-viral actions and roles in limiting excessive inflammatory responses) have been published. Observational studies clearly show that a suboptimal micronutrient status, particularly of vitamins D and C, zinc, and selenium, is associated with an increased risk of SARS-CoV infection, and higher severity of disease progression [7]. Good quality studies are required to draw solid conclusions, but available interventional studies on the effects of individual micronutrients, including vitamin D and zinc, suggest that they have a role in reducing the severity of COVID-19 [2]. In addition, adequate nutrition is important to support a robust immune response to vaccination and should not be overlooked, especially in higher-risk populations, such as older people, or those at risk of inadequate micronutrient status. The low intake of several micronutrients is known to impair vaccination responses, and must be considered in the context of COVID-19 vaccination programs [2,8].

The critical role of ensuring an adequate micronutrient status in COVID-19 mitigation, especially in at-risk groups (e.g., older age, concomitant polymorbidities, pregnant women), has increasingly been recognized in clinical guidelines (e.g., ESPEN, the European Society for Clinical Nutrition and Metabolism), and by health authorities (e.g., the World Health Organization). These have called for the prevention, diagnosis, and treatment of malnutrition in the management of COVID-19 patients to improve both short- and long-term prognosis [9,10]. Considering the widespread and global occurrence of micronutrient deficiencies, their impact on the immune system and resistance to infection [6], and the emerging science on COVID-19, internationally recognized experts in immune nutrition have requested immediate action from government authorities to support optimal micronutrient status in their strategies against COVID-19 [1,11].

However, it can be difficult to achieve optimal micronutrient intakes for immune defenses through a well-balanced and diverse diet alone [6]; particularly as micronutrients such as vitamins C and D need to be taken in doses higher than the recommended dietary allowance to be immune effective. In addition, hectic and stressful lifestyles and health conditions such as obesity and metabolic disorders (for example, diabetes) can create micronutrient imbalances and put additional strain on the immune system [1,6]. Therefore, the addition of a micronutrient supplement to a balanced daily diet is a safe, effective, and low-cost strategy to achieve optimal micronutrient status, support a well-functioning immune system, and enable a proper immune response to vaccination, important considerations during the COVID-19 pandemic, especially in certain groups of the population at risk.

The COVID-19 pandemic has highlighted the difficulty of restraining outbreaks of novel viruses and the associated public health burden. Supporting a healthy immune system by ensuring good nutrition, self-care and maintaining an adequate micronutrient intake is an important strategy in the battle to limit the spread and clinical impact of such highly-contagious viruses. Ensuring optimal micronutrient intakes, or at least addressing micronutrient insufficiencies, is of paramount importance to maximize immune protection of the population against the deleterious effect of SARS-CoV and to prime it for potential future pandemics. In parallel, more research is needed to further understand the effects of targeted micronutrient supplementation on the immune response, and thus on reducing the global burden of infection.

## Data Availability

Not applicable.
